# High Levels of miR-483-3p Are Present in Serum Exosomes Upon Infection of Mice With Highly Pathogenic Avian Influenza Virus

**DOI:** 10.3389/fmicb.2020.00144

**Published:** 2020-02-11

**Authors:** Tadashi Maemura, Satoshi Fukuyama, Yoshihiro Kawaoka

**Affiliations:** ^1^Division of Virology, Department of Microbiology and Immunology, The Institute of Medical Science, The University of Tokyo, Tokyo, Japan; ^2^Department of Pathobiological Sciences, School of Veterinary Medicine, University of Wisconsin-Madison, Madison, WI, United States; ^3^Department of Special Pathogens, International Research Center for Infectious Diseases, The Institute of Medical Science, The University of Tokyo, Tokyo, Japan

**Keywords:** influenza virus, exosome, miR-483-3p, innate immunity, vascular endothelial cells

## Abstract

Exosomes, the extracellular vesicles that contain functional proteins and RNAs, regulate cell-cell communication. Recently, our group reported that levels of various microRNAs (miRNAs) in bronchoalveolar lavage fluid exosomes were highly increased in influenza virus-infected mice and that one of those miRNAs, miR-483-3p, was involved in the potentiation of the innate immune responses to influenza virus infection in mouse type II pneumocytes. Here, we evaluated exosomal miR-483-3p levels in the serum of influenza virus-infected mice and found that miR-483-3p levels were significantly increased during infection with a highly pathogenic avian H5N1 influenza virus. Moreover, miR-483-3p-enriched exosomes derived from type II pneumocytes potentiated the expression of proinflammatory cytokine genes in vascular endothelial cells. Our findings suggest that serum exosomal transfer of miR-483-3p might be involved in the inflammatory pathogenesis of H5N1 influenza virus infection.

## Introduction

The emergence of highly pathogenic avian influenza (HPAI) viruses has raised serious concerns worldwide. HPAI viruses show strong pathogenicity in mammals and elicit dysregulation of proinflammatory cytokine production, which leads to multiple organ damage (Yuen et al., 1998; Cheung et al., 2002; Chan et al., 2005). However, the mechanism of cytokine dysregulation is not fully understood.

Exosomes are cell-derived extracellular vesicles (30–100 nm in diameter) (Raposo and Stoorvogel, 2013), containing functional proteins, mRNAs, and microRNAs (miRNAs). They function in cell-cell communication by being delivered to recipient cells (Valadi et al., 2007).

miRNAs are single-stranded non-coding RNAs that mediate the silencing of target mRNAs by binding to the complementary sequence in the 3′-untranslated region (UTR). Numerous reports indicate that altered miRNA expression is associated with pathogenicity in influenza virus infection (Li et al., 2010, 2011; Tambyah et al., 2013; Wu et al., 2013; Hsu et al., 2017). Recently, we reported that the miRNA miR-483-3p is involved in the innate immune response to influenza virus infection (Maemura et al., 2018). miR-483-3p was present at high levels in bronchoalveolar lavage fluid (BALF) exosomes in influenza virus-infected mice and potentiated *IFN-β* and inflammatory cytokine gene expression in type II pneumocytes upon influenza virus infection (Maemura et al., 2018). In addition to BALF, exosomes are present in most body fluids including serum (Patton et al., 2015). It has been reported that tissue-derived serum exosomes transfer and function in vascular endothelial cells (Tominaga et al., 2015; Di Modica et al., 2017; Yang et al., 2017). Moreover, the microvascular endothelium plays key roles in the regulation of the inflammatory response to influenza virus infection (Chan et al., 2009; Teijaro et al., 2011; Viemann et al., 2011; Ramos and Fernandez-Sesma, 2012). Inflammatory responses elicited by influenza virus infection in endothelial cells are mediated by activation of the NF-κB (Schmolke et al., 2009; Viemann et al., 2011; Ramos and Fernandez-Sesma, 2012). Because miR-483-3p has been reported to potentiate the activation of the transcription factors IRF3 and NF-κB in MLE-12 cells, we hypothesized that miR-483-3p could also potentiate the innate immune response in cells other than lung epithelial cells. However, it is not known whether miR-483-3p is present in serum exosomes in influenza virus-infected mice and whether miR-483-3p is involved in the immune response in the vascular endothelium during influenza virus infection.

In this study, we investigated the levels of serum exosomal miR-483-3p in influenza virus-infected mice and whether exosomal transfer of miR-483-3p affects the inflammatory response in vascular endothelial cells.

## Materials and Methods

### Cells

MILE SVEN 1 (MS1) cells, murine pancreatic islet endothelial cells, were purchased from American Type Culture Collection (ATCC, Manassas, VA, USA). MS1 cells were maintained in Dulbecco’s modified Eagle medium (Sigma-Aldrich, St. Louis, MO, USA, or ATCC) supplemented with 5% fetal calf serum (FCS) and penicillin-streptomycin solution. Human embryonic kidney 293 T (HEK293T) cells were maintained in Dulbecco’s modified Eagle medium supplemented with 10% FCS. NS1-expressing MDCK cells were a kind gift from Dr. Takeshi Ichinohe (Division of Viral Infection, Department of Infectious Disease Control, International Research Center for Infectious Diseases, Institute of Medical Science, the University of Tokyo) (Moriyama et al., 2016). NS1-expressing MDCK cells were maintained in MEM supplemented with 1% non-essential amino acids, and 10% FCS. Mouse lung epithelial (MLE)-12 cells were purchased from ATCC and maintained in DMEM/F-12 medium supplemented with 0.005 mg/ml insulin, 0.01 mg/ml transferrin, 30 nM sodium selenite, 10 nM hydrocortisone, 10 nM beta-estradiol, 10 mM HEPES, 2 mM L-glutamine, and 2% FCS. All cells were cultured at 37°C and 5% CO_2_.

### Plasmids

Viral RNAs (vRNAs) from influenza virus were isolated by using a QIAamp Viral RNA Mini Kit (QIAGEN, Hilden, Germany) according to the manufacturer’s instructions. To generate plasmids for the expression of vRNAs, cDNAs derived from vRNAs were cloned between the promoter and terminator sequences of RNA polymerase I, as described previously (Neumann et al., 1999). Plasmids for the expression of vRNAs encoding NS1 with mutations (R38A, K41A, E96A, and E97A) in the NS segments were generated by site-specific mutagenesis with PCR as described previously (Talon et al., 2000; Gack et al., 2009).

### Viruses

A/Puerto Rico/8/34 (H1N1; PR8) and NS1-mutant PR8 virus were generated by using reverse genetics using HEK293T cells (Neumann et al., 1999). Viruses were propagated in MDCK or NS1-expressing MDCK cells at 37°C for 48 h in MEM containing L-(tosylamido-2-phenyl) ethyl chloromethyl ketone-treated trypsin (0.8 μg/ml) and 0.3% bovine serum albumin. The avian influenza viruses A/Anhui/1/13 (H7N9; Anhui) (Watanabe et al., 2013) and A/Vietnam/1203/04 (H5N1; VN1203) were available in our laboratory. All experiments with avian influenza virus were performed under biosafety level 3+ conditions.

### Mice

Six-week-old female C57BL/6 mice (Japan SLC, Inc. Shizuoka, Japan) were intranasally infected with 50 μl of 10^5^ plaque-forming unit (PFU) of the indicated viruses per mouse. All animal experiments were performed in accordance with the regulations of the University of Tokyo Committee for Animal Care and Use and were approved by the Animal Experiment Committee of the Institute of Medical Science of the University of Tokyo (PA15-10).

### Exosome Isolation and Labeling

Exosomes from mouse sera were isolated by using Total Exosome Isolation (from serum) reagent (Thermo Fisher Scientific, Waltham, MA, USA). Exosomes from the media of cultured MLE-12 cells (4 × 10^5^) were isolated by using Total Exosome Isolation (from cell culture media) reagent (Thermo Fisher Scientific). The pellets containing the exosomes were resuspended in 100 μl of PBS. For labeling, the exosome-containing PBS was incubated with 10 μM SYTO RNASelect^™^ (Thermo Fisher Scientific) for 15 min at 37°C. To remove the unincorporated dye, Exosome Spin Columns (MW 3000) (Thermo Fisher Scientific) were used according to the manufacturer’s instructions. MS1 cells were incubated with labeled exosomes for the indicated times at 37°C. After being washed twice with PBS and fixed with 4% paraformaldehyde for 10 min at room temperature, the cells were treated with Hoechst 33342 (Thermo Fisher Scientific) for nuclei staining and were observed by use of a Nikon A1+ confocal laser microscope system.

### Immunofluorescence

Influenza virus-infected MS1 cells were washed three times with PBS and fixed with 4% paraformaldehyde for 10 min at room temperature. After permeabilization with PBS containing 0.1% Triton X-100, the cells were blocked with 3% BSA in PBS for 30 min at room temperature. Then, the cells were incubated with a rabbit polyclonal anti-influenza A NP antibody (PA5-32242, Thermo Fisher Scientific) for 1 h at room temperature. Goat anti-rabbit IgG (H + L) secondary antibody, Alexa Fluor 594 conjugate (A-11012, Thermo Fisher Scientific) was used as the secondary antibody. The cell nuclei were stained with Hoechst 33342 (Thermo Fisher Scientific), and the cells were observed by use of an EVOS FL Imaging System (Thermo Fisher Scientific).

### RNA Isolation

Total RNA from exosomes was isolated by using the miRNeasy Serum/Plasma Kit (QIAGEN). miRNeasy Serum/Plasma Spike-In Control (Cel-miR-39) (QIAGEN) was added to each exosome sample for normalization. Total RNA from cultured cells was isolated by using the Maxwell 16 LEV simply RNA purification kit (Promega, Madison, WI, USA).

### miRNA Transfection

Approximately 4 × 10^5^ MLE-12 cells or 2 × 10^5^ MS1 cells were transfected twice with 5 pmoles of the indicated miRNA by using the Lipofectamine RNAiMAX Reagent (Thermo Fisher Scientific) with a 24-h interval between transfections according to the manufacturer’s instructions. Twenty-four hours after the second transfection, the transfected cells were used for each assay. For the miRNA transfections, miRCURY LNA^™^ microRNA Mimics (mmu-miR-483-3p) and a miRNA Mimic Control (Negative Control 5) were purchased from EXIQON (Vedbaek, Denmark).

### Detection of miRNA and mRNA

The expression of miRNA and mRNA was assessed by using reverse transcription quantitative polymerase chain reaction (qRT-PCR). Taqman MicroRNA Assays (Applied Biosystems, Foster City, CA, USA) were used for reverse transcription and PCR for miRNAs. The Taqman MicroRNA RT Kit (Applied Biosystems) and Taqman Universal PCR Master Mix II, w/o UNG (Applied Biosystems) were used for the RT and qRT-PCR experiments, respectively. snoRNA202 or Cel-miR-39 was used for normalization.

Oligo dT primers and Superscript III^™^ Reverse transcriptase (Thermo Fisher Scientific) were used for cDNA synthesis of mRNA. qRT-PCR was performed with the THUNDERBIRD SYBR qPCR MIX (TOYOBO, Osaka, Japan). *GAPDH* was used for normalization. qRT-PCR was carried out on an ABI 7900HT Fast Real-Time System (Applied Biosystems) or a QuantStudio^™^ 6 Flex Real-Time PCR System (Appiled Biosystems). Primer sets for *IFN-β*, *IL-6*, *CCL2*, *TNF-α*, *GAPDH*, and *IL-10* are described elsewhere (Bosurgi et al., 2012; Maemura et al., 2018). Primer sets for *TGF-β* are as follows: 5′-ACTATTGCTTCAGCTCCACAGAG-3′(forward) and 5′-CCCAGACAGAAGTTGGCATGGTA-3′(reverse). A Ct value of more than 36.0 was considered as not detected.

### Statistical Analysis

Statistical analysis was performed by using JMP 13.0.0 software or GraphPad Prism 7. *P*-values were considered significant if they were less than 0.05. The statistical analysis method used and the number of biological replicates and technical replicates for each experiment are described in each figure legend.

## Results and Discussion

**Figure 1:**
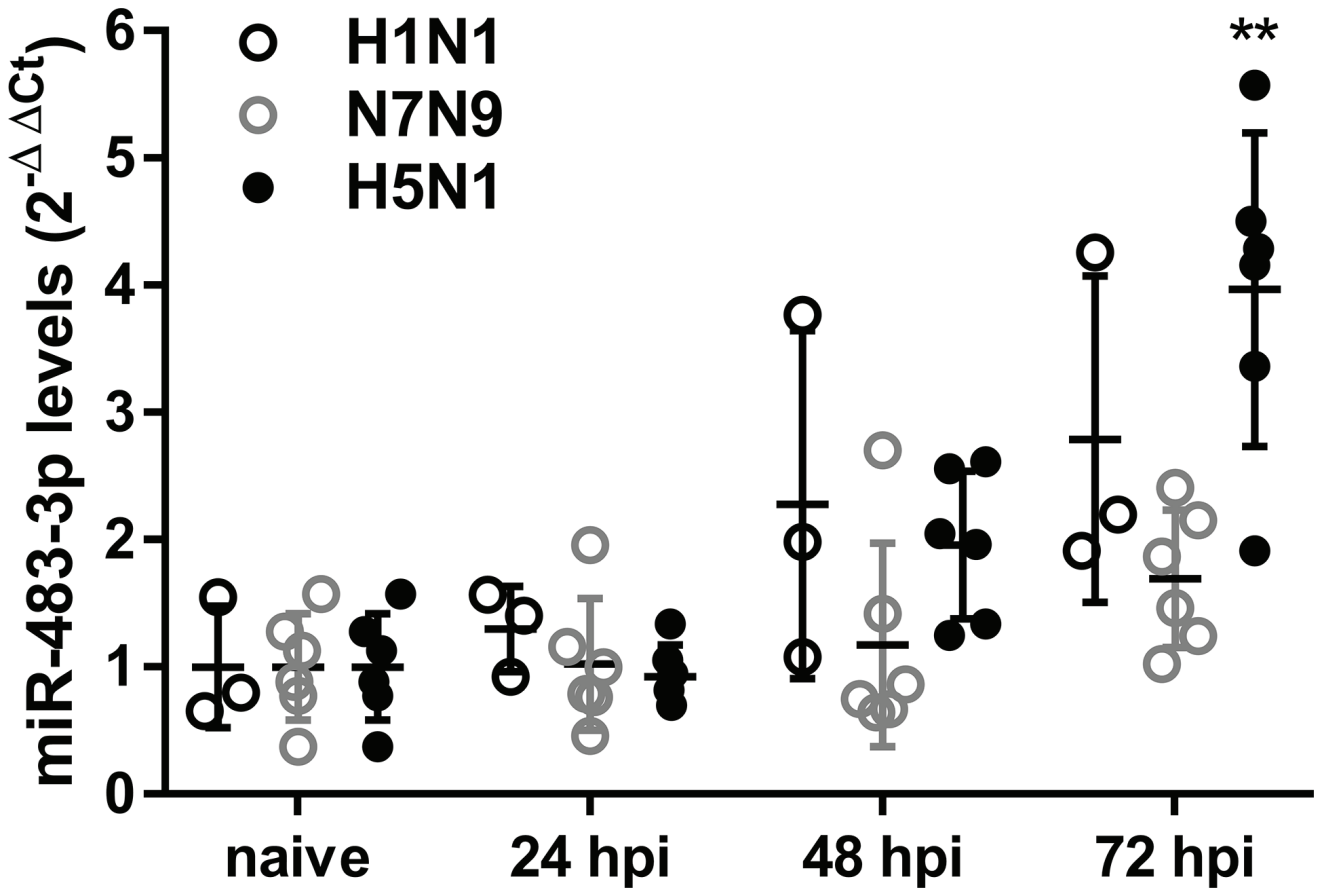
Serum exosomal miR-483-3p levels in influenza virus-infected mice. Mice were intranasally infected with 10^5^ pfu of influenza viruses (H1N1; PR8, H7N9; Anhui, or H5N1; VN1203). Sera were collected at 24, 48, and 72 hpi, and exosomal total RNA was isolated. The expression levels of miR-483-3p were quantified by qRT-PCR. Results are presented relative to those from naive mice (2^−ΔΔCt^). Statistical analysis was performed using a one-way ANOVA followed by Dunnett’s test. ^**^p < 0.01. There were six mice/group, except for H1N1; PR8, which had three mice/group. Data are presented as the mean ± SD.

To investigate levels of serum exosomal miR-483-3p, we intranasally infected mice with 10^5^ pfu of the following influenza virus strains of different pathogenicity: H1N1 [A/Puerto Rico/8/1934; PR8, 50% minimum lethal dose (MLD_50_), 10^3.5^ pfu], H7N9 (A/Anhui/1/2013; Anhui, MLD_50_, 10^3.5^ pfu), or H5N1 (A/Vietnam/1203/04; VN1203, MLD_50_, 3.3 pfu) (Watanabe et al., 2013; Fukuyama et al., 2015; Zhao et al., 2015). We collected blood at 24, 48, and 72 h post-infection (hpi) and isolated serum exosomes. Serum exosomes collected from naive mice served as a control. After isolation of total exosomal RNA, the presence of exosomal miR-483-3p in these sera was quantified by qRT-PCR. We found that serum exosomal miR-483-3p levels collected from H5N1-infected mice at 72 hpi were significantly higher than those from naive mice, but not H1N1- or H7N9-infected mice (Figure 1). Although there was no significant difference, the mean serum exosomal miR-483-3p levels in mice infected with H1N1 were higher at 48 and 72 hpi than the control levels. This result indicates that miR-483-3p in serum exosomes might be involved in the pathogenesis of highly pathogenic avian influenza virus infection.

Because we found an increased level of miR-483-3p in serum exosomes of mice infected with an H5N1 virus (Figure 1), we hypothesized that miR-483-3p-containing exosomes may function in vascular endothelial cells.

**Figure 2:**
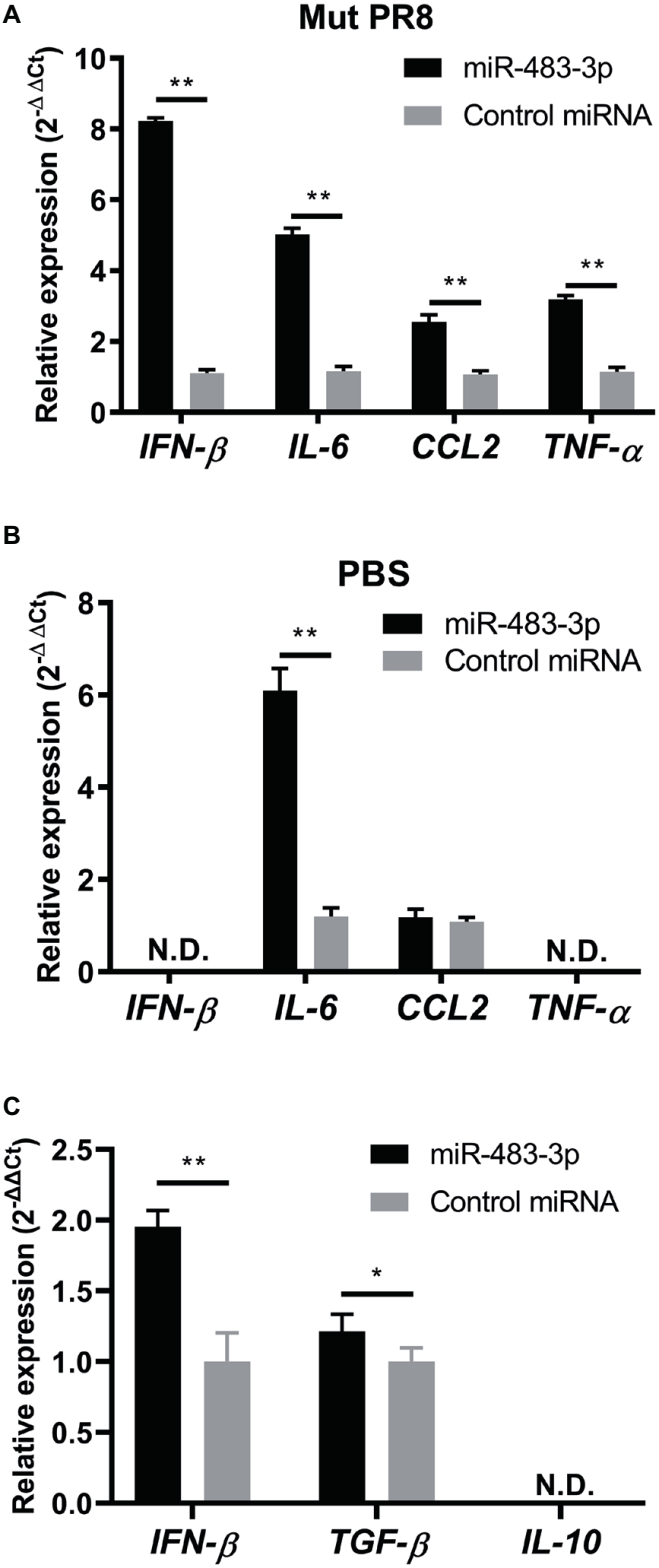
Proinflammatory and anti-inflammatory cytokine gene expression in MS1 cells upon miR-483-3p treatment. miRNA mimic treated MS1 cells were **(A)** infected with Mut-PR8 at an MOI of 0.2, or **(B)** treated with PBS for 12 h. The cells were then lysed and total RNA was isolated. *IFN-β*, *IL-6*, *CCL2*, and *TNF-α* gene expression was quantified by qRT-PCR. **(C)** miRNA mimic-treated MS1 cells were infected with Mut-PR8 at an MOI of 0.2 for 12 h. The cells were then lysed and total RNA was isolated. IFN*-β*, *TGF-β*, and *IL-10* gene expression was quantified by qRT-PCR. Results are presented relative to the control miRNA-transfected cell levels (2^−ΔΔCt^). Statistical analysis was performed by using Student’s t-test. ^**^p < 0.01, ^*^p < 0.05. There were **(A,B)** three technical replicates and **(C)** five technical replicates. qRT-PCR was carried out on **(A,B)** an ABI 7900HT Fast Real-Time System, or **(C)** a QuantStudio™ 6 Flex Real-Time PCR System. Data are presented as the mean ± SD.

To test this idea, first we evaluated whether transfection of miR-483-3p mimics potentiates the expression of *IFN-β* or proinflammatory cytokine genes (*IL-6*, *CCL2*, and *TNF-α*) in mouse endothelial cells (MS1 cells) by using qRT-PCR. Twenty-four hours after treatment with miR-483-3p mimics or control miRNA mimics, the MS1 cells were infected with NS1-mutated PR8 (Mut-PR8) virus for 12 h. This NS1 mutant contains four amino acid mutations, R38A, K41A, E96A, and E97A, which lead to activation of innate immune responses (Talon et al., 2000; Donelan et al., 2003; Gack et al., 2009). The R38A/K41A and E96A/E97A mutations potentiate innate immune responses *via* a defect in dsRNA binding activity and negating the interaction of NS1 with TRIM25, respectively (Talon et al., 2000; Donelan et al., 2003; Gack et al., 2009). Because Mut-PR8 induced stronger innate immune responses than did wild-type PR8 (data not shown), we used this virus for this experiment. MS1 cells were efficiently infected with Mut-PR8 viruses (Supplementary Figure S1). We found that miR-483-3p treatment potentiated the gene expression of *IFN-β*, *IL-6*, *CCL2*, and *TNF-α* in Mut-PR8-infected cells (Figure 2A). Next, to test whether the activation of cytokine expression is triggered by miR-483-3p independently of viral infection, we treated the MS1 cells with PBS after miRNA mimic transfection and evaluated the proinflammatory cytokine gene expression. We found that miR-483-3p potentiated IL-6 expression in MS1 cells in the absence of virus infection (Figure 2B), suggesting that miR-483-3p *per se* might potentiate proinflammatory cytokine expression. In addition to proinflammatory cytokine genes, we also investigated the expression levels of the *TGF-β* and *IL-10* genes, which are known anti-inflammatory cytokines, upon miR-483-3p transfection in Mut-PR8-infected cells. We found weak potentiation of *TGF-β* gene expression by miR-483-3p transfection, whereas *IL-10* was not detected in MS1 cells (Figure 2C). The weak potentiation of *TGF-β* and undetectable levels of *IL-10* in MS1 cells suggest that proinflammatory responses are the major responses upon miR-483-3p transfection in vascular endothelial cells infected with Mut-PR8 viruses, rather than anti-inflammatory responses. These results indicate that miR-483-3p elicits proinflammatory cytokine induction in MS1 cells during influenza virus infection.

**Figure 3:**
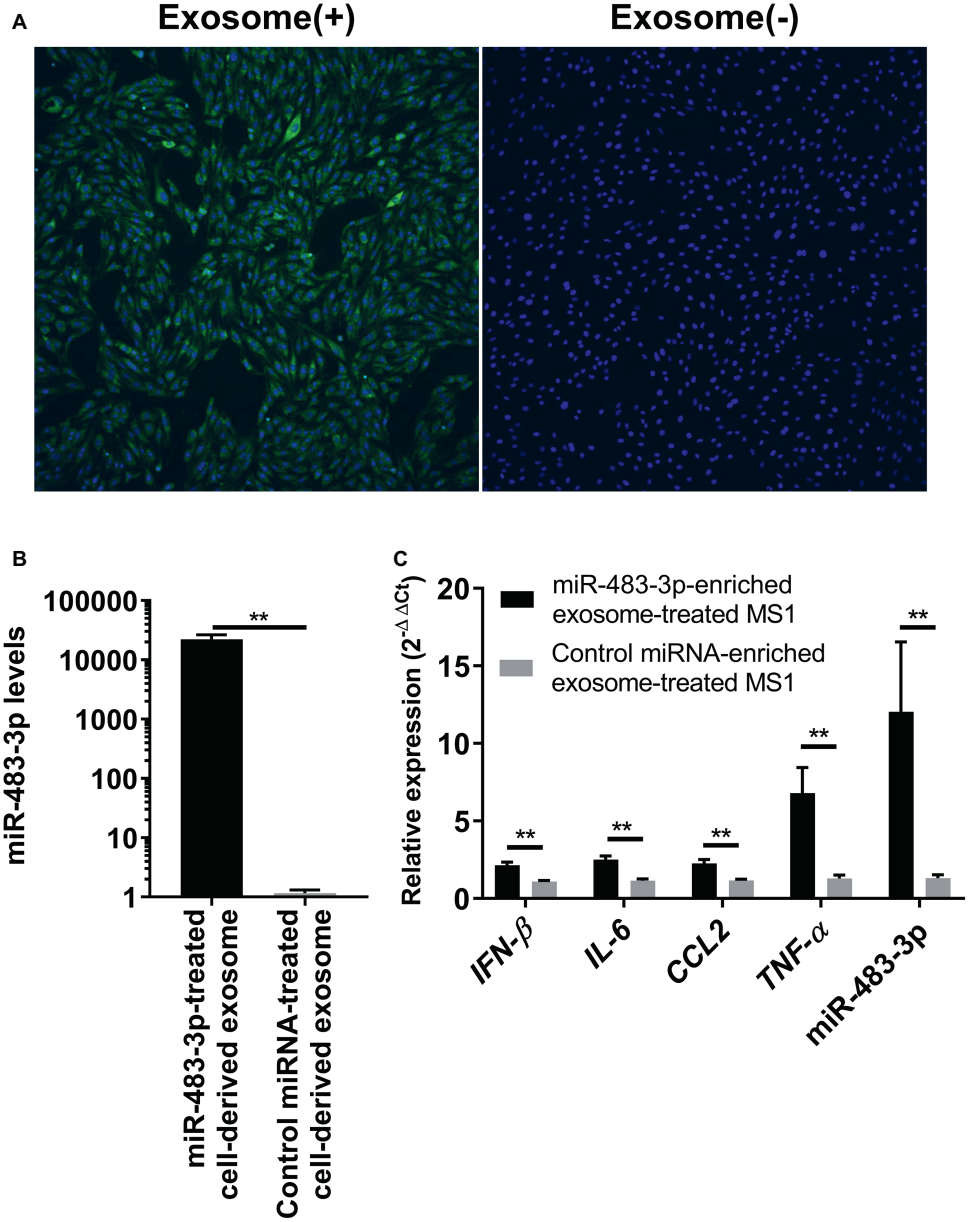
Uptake of miR-483-3p-enriched exosomes by MS1 cells potentiates proinflammatory cytokine gene expression. **(A)** MS1 cells were incubated for 6 h with exosomes derived from MLE-12 cells. MLE-12 cell-derived exosomes or the same volume of PBS was labeled with SYTO RNASelect and internalization of exosomes was evaluated by means of fluorescence confocal microscopy. **(B)** MLE-12 cells were transfected with miR-483-3p or a control miRNA mimic. At 24 h post-transfection, the culture medium was changed, and the cells were incubated for an additional 48 h. Exosomes were isolated from the culture media and total exosomal RNA was isolated. The expression levels of miR-483-3p were quantified by qRT-PCR. Results are presented relative to those from Control miRNA mimic-transfected cells (2^−ΔΔCt^). **(C)** Total exosomes were isolated from the culture media of MLE-12 cells transfected with miR-483-3p or Control miRNA mimic and were resuspended in PBS. Exosomes were added to the culture media of MS1 cells and incubated for 48 h. MS1 cells were then infected with Mut-PR8 and total RNA was isolated from the cells at 12 hpi. The expression levels of *IFN-β*, *IL-6*, *CCL2*, *TNF-α*, and miR-483-3p were quantified by qRT-PCR. Results are presented relative to those from MS1 cells cultured with Control miRNA-enriched exosomes (2^−ΔΔCt^). Statistical analysis was performed by using Student’s *t*-test. ^**^p < 0.01. There were six technical replicates in each experiment. Data are presented as the mean ± SD.

Because our previous study showed that BALF exosomal miR-483-3p levels are highly increased in H5N1 virus-infected mice (Maemura et al., 2018), we next examined whether the exosomes obtained from lung epithelial cells could transfer the miRNA to vascular endothelial cells. We developed an *in vitro* direct exosome transfer model using MLE-12 cells (epithelial cells) and MS1 cells (endothelial cells). We cultured MS1 cells with fluorescent-labeled exosomes for 6 h. Then, we evaluated the uptake of the exosomes by using confocal microscopy. The fluorescent-labeled exosomes were efficiently internalized by the MS1 cells (Figure 3A), suggesting that exosomes released from lung epithelial cells can be transferred into the vascular endothelium.

Next, we evaluated whether exosome-transferred miR-483-3p could potentiate the expression of proinflammatory cytokine genes in vascular endothelial cells upon virus infection. To obtain exosomes that contained sufficient miR-483-3p, we collected the culture medium of MLE-12 cells transfected with the miR-483-3p mimic. The culture medium of control miRNA-transfected cells served as a control. The levels of miR-483-3p in the exosomes isolated from the culture medium of the miR-483-3p mimic-transfected MLE-12 cells were significantly increased (Figure 3B). We then cultured MS1 cells for 48 h with exosomes containing miR-483-3p and subsequently infected the cells with Mut-PR8 virus for 12 h. We performed qRT-PCR and found that the expression of *IFN-β*, proinflammatory cytokines, and miR-483-3p were all upregulated in miR-483-3p-enriched exosome-treated MS1 cells (Figure 3C). These results suggest that exosomal transfer of miR-483-3p might induce high levels of cytokines in vascular endothelial cells during HPAI virus infection.

In this study, we evaluated the contribution of exosomal miR-483-3p to the inflammatory responses in endothelial cells during influenza virus infection. We found that serum exosomes from H5N1 virus-infected mice contained significantly higher amounts of miR-483-3p than the control (Figure 1) and that miR-483-3p-enriched MLE-12 cell-derived exosomes are internalized into MS1 cells where they potentiate inflammatory cytokine gene expression (Figures 3A,C).

We used two methods to evaluate the potentiation of innate immune responses by miR-483-3p: a transfection experiment and a direct exosome transfer model. The expression levels of *IFN-β* and inflammatory cytokine genes differed between the MS1 cells transfected with miR-483-3p mimics (Figure 2) and cells incubated with miR-483-3p-enriched exosomes (Figure 3A). Although we do not know the exact reasons why exosome-mediated transfer of miR-483-3p in MS1 cells potentiated inflammatory cytokine gene expression to a greater extent that did miR-483-3p transfection, we can offer two possible explanations. First, the amount of miR-483-3p in MS1 cells differed between these two experimental approaches. Second, the exosomes collected from MLE-12 cells may have contained other RNAs and proteins. These differences may explain the observed differences.

To evaluate the function of serum exosomal miR-483-3p, we attempted to isolate serum exosome from the mice infected with H5N1 virus and incubate them with MS1 cells. However, because it was not possible to isolate serum exosomes from virus-infected mice without virus contamination, we collected exosomes derived from lung epithelial cells (MLE-12 cells) transfected with miR-483-3p mimics and evaluated their effect on endothelial cells (MS1 cells) *in vitro*. Further study is required to investigate the direct roles of miR-483-3p-containing exosomes in the serum of H5N1-virus infected mice.

Our studies indicate that exosomes mediate gene regulation between lung epithelial cells and vascular endothelial cells. Vascular endothelial cells play important roles in the host responses to influenza virus infection (Chan et al., 2009; Teijaro et al., 2011; Viemann et al., 2011). H5N1 HPAI virus elicits a strong NF-κB-dependent cytokine response in endothelial cells compared with that elicited by the PR8 strain (Viemann et al., 2011). Although miR-483-3p has previously been shown to potentiate the activation of NF-κB in MLE-12 cells (Maemura et al., 2018), we did not perform experiments to evaluate miR-483-3p-mediated NF-κB activation in MS1 cells in the present study. Further study is required to elucidate the molecular mechanism of the potentiation of innate immune responses by miR-483-3p. Our results suggest a novel regulatory mechanism of inflammation *via* endothelial cells mediated by exosomal miRNA during influenza virus infection.

In conclusion, our study revealed that miR-483-3p is increased in serum exosomes in HPAI virus-infected mice and that exosome-mediated transfer of miR-483-3p induces the upregulation of inflammatory cytokines in vascular endothelial cells. Exosome-mediated miR-483-3p transfer might be one of the mechanisms by which excessive secretion of proinflammatory cytokines occurs upon HPAI virus infection.

## Data Availability Statement

The datasets generated for this study are available on request to the corresponding author.

## Ethics Statement

All animal experiments were performed in accordance with the regulations of the University of Tokyo Committee for Animal Care and Use and were approved by the Animal Experiment Committee of the Institute of Medical Science of the University of Tokyo (PA15-10).

## Disclosures

YK is a Co-Founder of FluGen. He has received grant support from Chugai Pharmaceuticals, Daiichi Sankyo Pharmaceutical, Toyama Chemical, Tauns Laboratories, Inc., Denka Seiken Co. Ltd., Ttsumura and Co; and royalties from MedImmune. YK holds the following patents: recombinant influenza viruses for vaccines and gene therapy (US Patent Number: US 8715940 B2); viruses comprising mutant ion channel protein (US Patent No. 6872395); mutant cells with altered sialic acid (US Patent No. 7176021); filovirus vectors and noninfectious filovirus-based particles (US Patent No. 7211378); signal for packaging of influenza virus vectors (US Patent No. 7585657B2); viruses encoding mutant membrane protein (US Patent No. 7588769B2); recombinant influenza vectors with a polII promoter and ribozymes for vaccines and gene therapy (US Patent No. 7723094); recombinant influenza vectors with tandem transcription units (US Patent No. 7968101B2); adenoviral vectors for influenza virus production (US Patent No. 8043856); viruses comprising mutant ion channel protein (US Patent No. 8057806); cell-based systems for producing influenza vaccines (US Patent No. 8163523); influenza B viruses with reduced sensitivity to neuraminidase inhibitor (US Patent No. 8465960); high titer recombinant influenza viruses for vaccine and gene therapy (U.S. Patent No. 8475806 B2); influenza A virus with attenuating mutations in NS2 protein (U.S. Patent No. 8507247); and neuraminidase-deficient live influenza vaccines (U.S. Patent No. 8597661).

## Author Contributions

TM, SF, and YK designed the study, analyzed the data, and wrote the manuscript. TM and SF performed the experiments. YK oversaw the project.

### Conflict of Interest

The authors declare that the research was conducted in the absence of any commercial or financial relationships that could be construed as a potential conflict of interest.

## Supplementary Material

SUPPLEMENTARY FIGURE 1 Evaluation of infectivity of MS1 cells with Mut-PR8 virus. MS1 cells were infected with Mut-PR8 at different MOIs (MOI = 1, 0.1, and 0.01) for 24 h. MS1 cells were incubated with anti-NP antibodies and secondary antibodies conjugated with Alexa 594. Cell nuclei were stained with Hoechst 33342, and the cells were evaluated by using fluorescence microscopy.
