# Supplementary figures and images for: High Levels of miR-483-3p Are Present in Serum Exosomes Upon Infection of Mice With Highly Pathogenic Avian Influenza Virus
Source: Front Microbiol. 2020 Feb 11;11:144. doi: 10.3389/fmicb.2020.00144 (PMC7026002; doi:10.3389/fmicb.2020.00144)

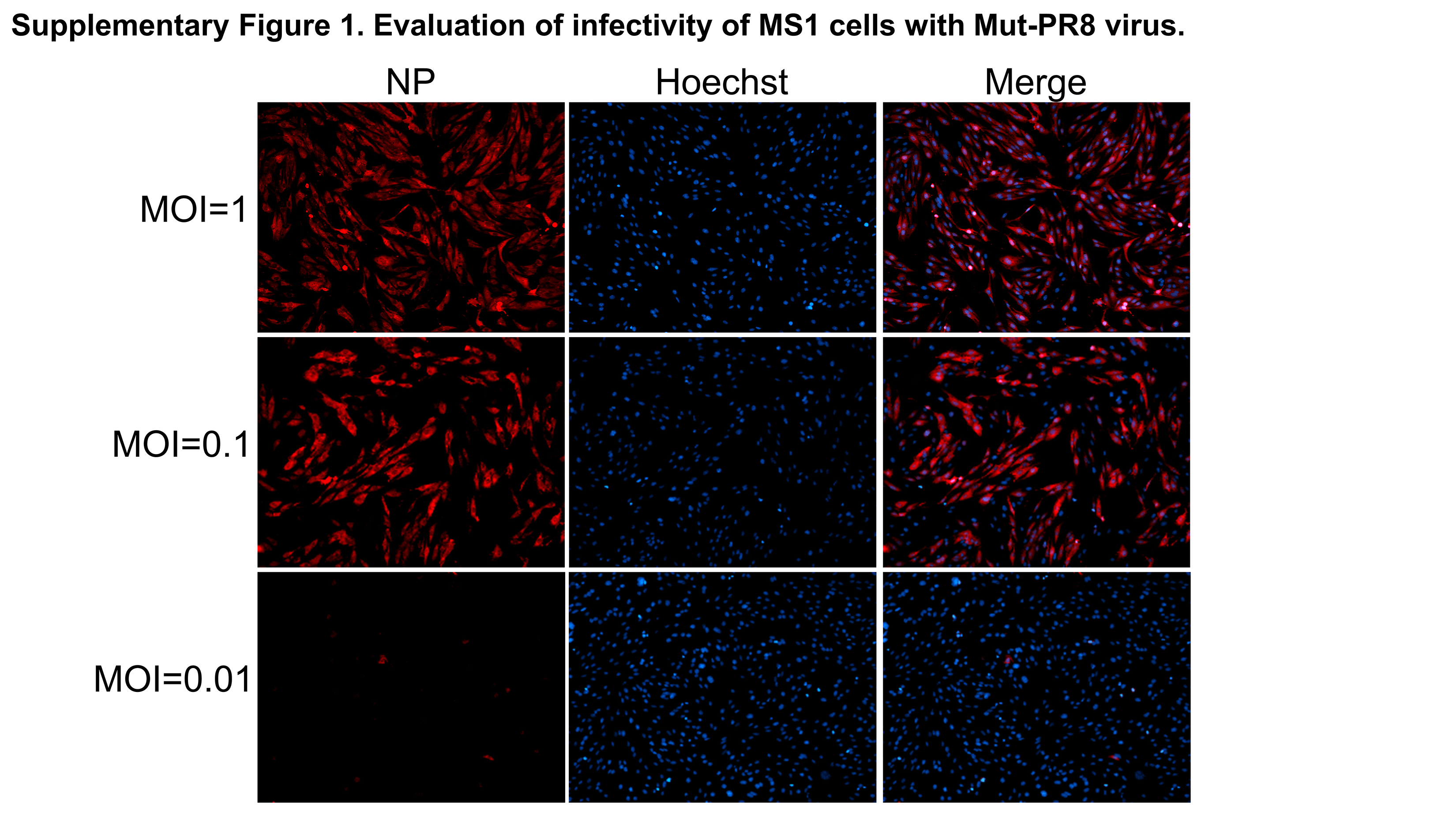

Supplement: SUPPLEMENTARY FIGURE 1 — Evaluation of infectivity of MS1 cells with Mut-PR8 virus. MS1 cells were infected with Mut-PR8 at different MOIs (MOI = 1, 0.1, and 0.01) for 24 h. MS1 cells were incubated with anti-NP antibodies and secondary antibodies conjugated with Alexa 594. Cell nuclei were stained with Hoechst 33342, and the cells were evaluated by using fluorescence microscopy. [file Image_1.tif]
